# Physiological Mechanisms of Silicon-Induced Drought Tolerance in Crops

**DOI:** 10.3390/plants15172721

**Published:** 2026-09-05

**Authors:** Anshu Rastogi

**Affiliations:** Department of Bioclimatology, Poznań University of Life Sciences, 60-649 Poznań, Poland; anshu.rastogi@up.poznan.pl

**Keywords:** silicon, drought stress, abiotic stress tolerance, aquaporin, osmotic adjustment, antioxidant defence, hormonal signalling, crop physiology

## Abstract

Drought is one of the most damaging abiotic stresses affecting global crop productivity, and its frequency and severity are projected to increase under ongoing climate change. Silicon (Si), although not classified as an essential nutrient, is increasingly regarded as a “quasi-essential” beneficial element that improves crop performance under water-limited conditions. This review summarises the physiological mechanisms of Si-induced drought tolerance, based mainly on literature published in the past five years. Rather than presenting these mechanisms as an inventory of separate physiological effects, the review reframes them as a coordinated stress-tolerance network linked by shared transcriptional regulation, and it organises the evaluation around three conceptual tensions that remain unresolved in the literature: the opposite direction of Si’s effect on transpiration, the extent to which Si-accumulating grasses and Si-excluding dicots rely on equivalent mechanisms, and the non-linearity of dose responses. Si uptake and transport via Lsi1, Lsi2, and Lsi6, and the resulting difference between Si-accumulating and Si-excluding species, are discussed together with the enhancement of root growth and aquaporin-mediated hydraulic conductance; stomatal and photosynthetic regulation; osmotic adjustment through compatible solute accumulation; enzymatic and non-enzymatic antioxidant defence; hormonal signalling involving abscisic acid, jasmonic acid, ethylene, and auxin; reinforcement of cell walls and vascular tissue; and the transcriptional networks coordinating these responses. Si’s influence on rhizosphere nutrient dynamics and the dependence of its efficacy on genotype, dose, and application method are also considered. A consolidated mechanistic scheme is presented, showing how these pathways converge on a drought-tolerant phenotype characterised by sustained growth, improved water-use efficiency, and faster recovery. Future research priorities, including field validation, standardisation of application protocols, multi-omics integration, and Si–microbiome interactions, are outlined to support the translation of these mechanistic insights into practical drought-management strategies.

## 1. Introduction

Drought remains one of the most damaging abiotic stresses in global agriculture, and both its frequency and severity are projected to increase as climate change progresses [1,2]. Water deficit disrupts plant function at almost every level, from stomatal closure and reduced CO_2_ assimilation to oxidative damage of membranes and proteins, and it constrains crop yield and quality across major cereal- and legume-producing regions worldwide [3]. Silicon (Si), though not classified as an essential nutrient, is now regarded as a “quasi-essential” beneficial element that consistently improves crop performance under water-limited conditions [4,5,6,7]. Its benefits are not confined to a single pathway. They emerge instead from an interconnected set of morphological, physiological, biochemical, and molecular adjustments that together let plants sustain water status, protect the photosynthetic apparatus, and limit oxidative injury when soil moisture is limited. Yet this evidence has largely been presented as a list of separate physiological effects, leaving unclear whether it reflects a single coordinated response system and why several of the underlying findings conflict with one another. This article synthesises the principal physiological mechanisms through which Si mediates drought tolerance in crop plants and presents a consolidated mechanistic scheme showing how these pathways interact to produce an integrated drought-tolerant phenotype.

Beyond compiling this evidence, the review makes two further contributions. Si-induced drought tolerance is reframed here not as a set of independent physiological effects but as a coordinated stress-tolerance network, in which root hydraulics, photochemistry, osmotic adjustment, redox balance, hormonal signalling, and cell-wall reinforcement are mechanistically coupled through shared transcriptional regulators; this framework has not previously been made explicit in single-mechanism or single-species reviews. Three conceptual tensions that the current literature has largely left unresolved are also identified: (i) why Si alters transpiration in opposite directions across studies despite a consistent improvement in water-use efficiency; (ii) whether the benefit conferred on Si-accumulating grasses is mechanistically equivalent to that conferred on Si-excluding dicots, or fundamentally distinct from it; and (iii) whether the concentration-dependent, often non-linear response to Si reflects a genuine physiological optimum or an artefact of inconsistent experimental design. These tensions are treated here as the central problem around which the review is organised, and they motivate the critical evaluation and research agenda presented below.

Recent syntheses in this space, including [5], have made valuable contributions by comprehensively compiling and integrating Si–drought mechanisms from physiology to the molecular level, in that case tracing how the field’s methodological focus has shifted over time from transporter identification toward multi-omics approaches. This review’s specific contribution is distinct: rather than a further inventory of Si–drought mechanisms, it reframes the evidence as a coordinated stress-tolerance network and organises it around three conceptual tensions that are used throughout to interrogate, rather than simply summarise, the evidence.

## 2. Silicon Uptake, Transport, and Species-Dependent Accumulation

**Figure 1:**
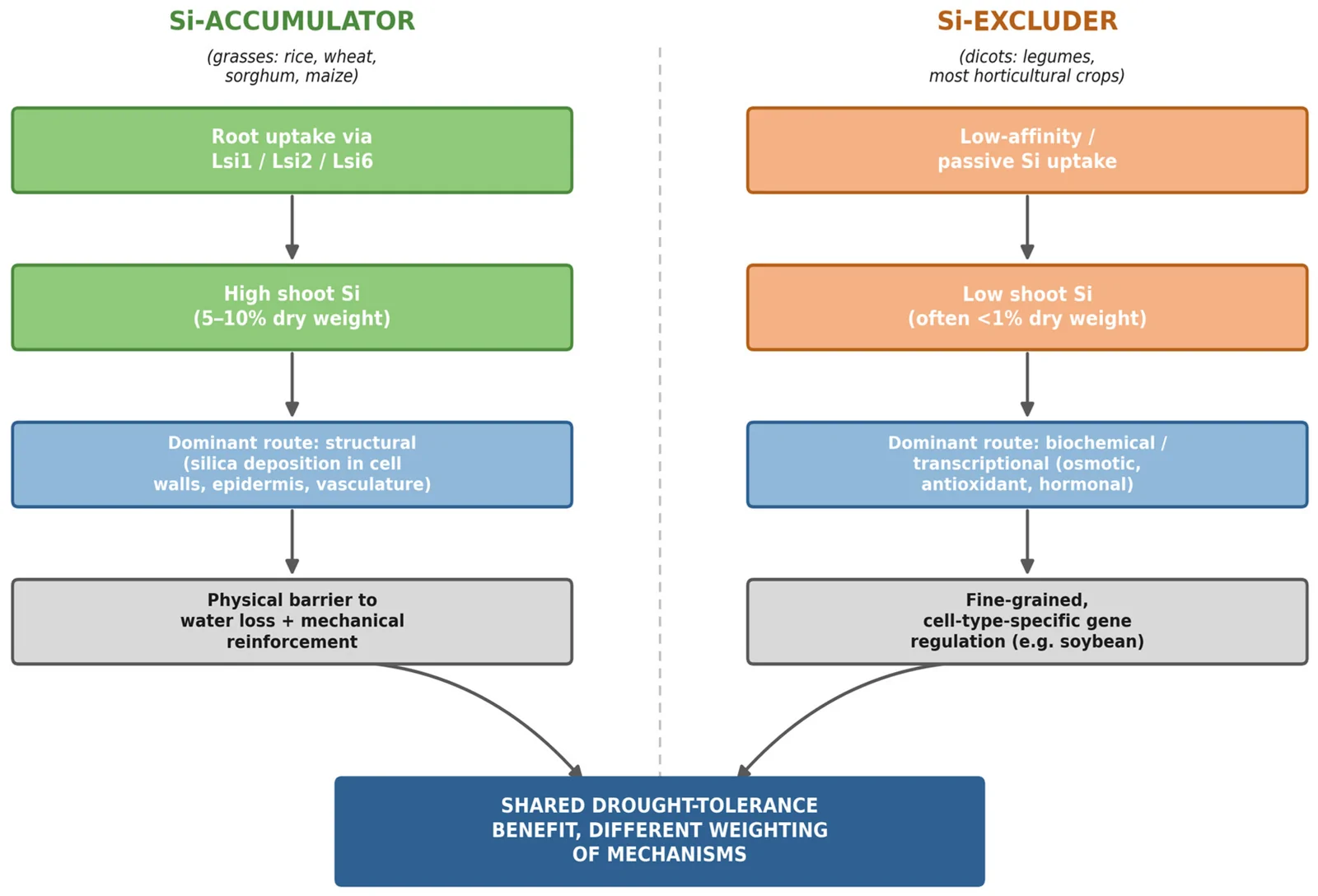
Comparison of Si-accumulator (grasses) and Si-excluder (dicot) crop species, showing their contrasting uptake routes and dominant mechanistic strategies for drought protection.

Si is absorbed by roots as monosilicic acid, Si(OH)_4_, through influx and efflux transporters, most notably Lsi1 and Lsi2, and is subsequently distributed to shoots via the transpiration stream, with Lsi6 additionally unloading Si into aerial tissues [8,9]. Lsi1 belongs to the nodulin-26-like intrinsic protein (NIP) subfamily of aquaporins and possesses a selectivity filter that allows silicic acid, rather than water alone, to permeate the channel, while Lsi2 acts as a proton-coupled efflux antiporter that moves Si from the root cortex into the stele against its concentration gradient [9,10]. The identification of Lsi1 as the first molecularly characterised silicon transporter, achieved through positional cloning in rice, established the foundation on which this transport model has since been built [11]. Whether transporter expression itself is drought-responsive, rather than a fixed background process, is only partly resolved; a small number of studies report altered Lsi1 and Lsi2 transcript abundance under water deficit, and whether this feedback loop between drought signalling and Si-transporter regulation is adequately captured in current uptake models remains an open question. Species differ markedly in their capacity to accumulate Si. Grasses such as rice, wheat, sorghum, and maize are strong accumulators, depositing 5–10% of shoot dry weight as silica, whereas many dicots, including most legumes, take up comparatively little Si, often less than 1% of dry weight, yet still derive measurable stress-tolerance benefits [12]. Si’s protective effects are therefore not solely a function of bulk tissue accumulation; both accumulator and non-accumulator species appear to access at least some of the mechanisms discussed below, though to different extents and through different balances of structural and biochemical routes [13]. This apparent equivalence, which bears directly on tension (ii) raised in the Introduction, currently rests on comparatively thin quantitative ground for non-accumulators: direct dose-response and mechanistic data in Si-excluding legumes remain limited to a handful of species, among them lentil, chickpea, common bean, mung bean, and black gram, and even there the depth of transcriptional and physiological characterisation available is far sparser than for the well-studied accumulator grasses [14,15,16,17,18]. These contrasting strategies are summarised in Figure 1.

## 3. Root Growth, Aquaporin Regulation, and Water Uptake

Si consistently enhances root system function under drought. Si supplementation has been shown to increase root length, surface area, and branching density, thereby expanding the volume of soil from which water can be extracted, in wheat, sorghum, lentil, and oilseed rape [19,20,21]. At the cellular level, Si upregulates aquaporin genes of the plasma-membrane intrinsic protein (PIP) family; in sorghum, Si enhances expression of SbPIP1;6, SbPIP2;2, and SbPIP2;6, increasing root hydraulic conductance and water transport capacity under osmotic stress [22,23]. It is worth noting that this aquaporin response in sorghum was originally demonstrated under salt-induced rather than drought-induced osmotic stress [22]; the extrapolation to drought is plausible given the shared osmotic component of the two stresses, but it has not been directly confirmed under water deficit in that species. A similar aquaporin-mediated increase in root hydraulic conductance has been reported in tomato, where Si application simultaneously reduced membrane oxidative damage, suggesting that improved hydraulic function and reduced oxidative injury to membrane transport proteins are mechanistically linked [24]. These gains in hydraulic conductance are not universal, however; several studies report that Si supplementation leaves root hydraulic conductance unchanged, or even reduces it, under severe or prolonged water deficit, an outcome attributed to Si-induced apoplastic barrier formation restricting radial water flow once deposition becomes extensive, so the net effect likely depends on stress severity, duration, and the balance between aquaporin-mediated and apoplastic pathways of water movement. Greater root architecture combined with more efficient water channelling allows Si-treated plants to sustain tissue hydration for longer during progressive drought and underlies much of the downstream physiological protection described in later sections.

## 4. Stomatal Regulation and Photosynthetic Maintenance

Photosynthesis is highly sensitive to water deficit because stomatal closure, imposed to limit transpirational water loss, simultaneously restricts CO_2_ entry into the leaf. Si has been shown to modulate stomatal behaviour in ways that balance water conservation against carbon gain, but its effect on transpiration is inconsistent across the literature. Some field and glasshouse studies report reduced transpiration accompanied by improved water-use efficiency, whereas others report increased transpiration, again with improved water-use efficiency, depending on species, drought-imposition method, and Si application route [25,26]. A meta-analysis found no consistent effect of Si on stomatal conductance or transpiration in unstressed plants, but a significant increase in stomatal conductance specifically in drought-stressed plants, indicating that Si’s stomatal effects are context-dependent rather than fixed [27]. This meta-analytic picture is distinct from, though broadly consistent with, more recent single-study evidence: a multi-genotype wheat trial found that the direction and magnitude of Si’s effect on stomatal conductance depended strongly on cultivar and stress intensity rather than following one consistent rule, underscoring that genotype-level variation, and not only species-level differences, contributes to the apparent contradiction [28]. Even taken together, however, this evidence explains only part of the pattern; the transpiration paradox identified as tension (i) above is better described as partially clarified than resolved. Regardless of the direction of change in transpiration, Si consistently improves water-use efficiency and helps maintain chlorophyll content, photosystem II efficiency, and gas-exchange parameters such as net photosynthesis and stomatal conductance under drought [29]. Si has also been shown to preserve thylakoid membrane protein complexes, including components of photosystem I and II core complexes, thereby protecting the structural basis of the photosynthetic electron transport chain during water deficit [29]. A recent transcriptomic and physiochemical study in maize linked Si-induced drought resilience to coordinated shifts in jasmonic-acid signalling components that indirectly help preserve photosynthetic capacity under water deficit [30]. In the pseudocereal common buckwheat (*Fagopyrum esculentum* Moench), foliar Si application has similarly been shown to support photosynthetic electron transport, as reflected in chlorophyll-fluorescence-derived energy-flux parameters, and to aid post-stress recovery under combined drought and waterlogging conditions [31,32].

## 5. Osmotic Adjustment and Compatible Solute Metabolism

Maintaining cell turgor under declining soil water potential depends on the accumulation of compatible solutes that lower cellular osmotic potential without disrupting normal metabolism. Si has been shown to promote osmotic adjustment by supporting the accumulation of soluble sugars and free amino acids, and, in many, though not all, studies, proline, thereby sustaining water uptake and cellular turgor under drought [10,33]. Several studies report that Si application moderates excessive proline accumulation relative to drought-stressed controls, an observation interpreted as evidence that Si reduces the underlying severity of stress rather than simply amplifying a generic stress-osmolyte response [12]. Transcriptomic work in lentil, a Si-excluding legume, showed that Si supplementation downregulated proline biosynthesis and metabolism genes under drought while upregulating broader osmotic-stress-response genes, consistent with feedback inhibition of proline synthesis once cellular proline pools have accumulated [12]. In sorghum, Si-mediated osmotic adjustment in roots has also been linked to decreased root osmotic potential and increased leaf relative water content, supporting a coordinated root-to-shoot osmotic strategy [6]. A recurring limitation across this literature is that most studies report absolute osmolyte concentrations in Si-treated versus untreated drought-stressed plants without partitioning how much of the change is attributable to Si acting on osmolyte metabolism specifically, as opposed to Si simply lessening the severity of water deficit experienced by the plant; disentangling these two contributions would require dose-response designs paired with independent measures of plant water status, which are rarely reported.

## 6. Antioxidant Defence and Redox Homeostasis

Drought increases the production of reactive oxygen species (ROS), including superoxide anion radical and hydrogen peroxide, which damage lipids, proteins, and nucleic acids and can impair photosynthetic and membrane function. Si has been shown to enhance the activity of enzymatic antioxidants such as superoxide dismutase, catalase, peroxidase, and ascorbate peroxidase, and to support non-enzymatic antioxidants including glutathione and ascorbate [34,35]. This strengthened antioxidant capacity translates into measurable reductions in lipid peroxidation markers; Si reduced malondialdehyde and hydrogen peroxide accumulation by 42% and 12%, respectively, in drought-stressed soybean while increasing water-use efficiency by 25% [36]. A recent study in rice similarly showed that Si enhanced tolerance to combined drought and blast disease stress partly by modulating ROS accumulation and the expression of associated stress-responsive genes [37]. Si appears to act on redox-sensitive signalling pathways and transcription factors that regulate ROS-scavenging networks, and may additionally stabilise chloroplast and mitochondrial membranes, thereby limiting the sites of ROS generation [38]. This literature, however, tends to catalogue antioxidant enhancement as a uniformly positive outcome without systematically examining its dose- and species-dependency, or the theoretical possibility that sustained upregulation of antioxidant machinery under prolonged stress could tip redox balance toward an over-reduced state that itself impairs signalling; direct tests of this possibility in Si-treated, drought-stressed crops are still lacking. The picture is also incomplete because most of the studies discussed here quantify individual enzyme activities or end-point ROS markers rather than engaging with the broader biosynthetic and signalling logic connecting reactive oxygen and reactive nitrogen species; a recent synthesis of ROS/RNS biosynthesis and subcellular signalling across abiotic and biotic stress offers a more integrative framework into which Si-modulated redox homeostasis could usefully be situated [39]. By limiting oxidative damage to membranes and the photosynthetic apparatus, Si helps preserve cellular structure and function during water deficit, and this antioxidant protection is closely linked to the aquaporin- and hormone-mediated mechanisms described above and below.

## 7. Hormonal Regulation and Signalling Crosstalk

Si does not act as a simple stimulant of stress hormones but instead appears to fine-tune hormonal homeostasis in a way that balances defence against continued growth. Under drought, Si has been shown to moderate abscisic acid (ABA) accumulation to a level sufficient for stomatal regulation without imposing excessive growth inhibition, an effect linked to changes in the expression of ABA biosynthetic and catabolic genes [12,40]. The molecular basis of this hormonal recalibration is still described mostly at the level of correlated gene-expression changes rather than established mechanism; it remains unclear whether Si acts upstream by altering hormone transporter expression and polar transport, by modulating the activity of key biosynthetic and catabolic enzymes, or by changing receptor sensitivity and downstream signalling components, and these possibilities are not mutually exclusive. Si also influences jasmonic acid (JA), ethylene (ET), auxin, cytokinin, and gibberellin signalling, generally downregulating JA- and ET-related biosynthetic genes while upregulating auxin- and polyamine-related metabolism, a pattern interpreted as delaying senescence while supporting continued root and shoot growth under stress [6,12]. In peach seedlings, Si-enhanced drought resistance was attributed to coordinated regulation of stress-hormone synthesis and signal transduction alongside amino acid and sugar metabolism [41]. In maize, Si-mediated drought resilience has been linked to jasmonic-acid pathway components, including JAZ-repressor dynamics that regulate downstream stress-responsive transcription factors such as MYC2 [30]. How these hormonal pathways interact with one another under the combined pressure of Si supplementation and drought, rather than being considered one at a time, is not yet well resolved; ABA, JA, ethylene, and auxin signalling are extensively cross-regulated even without Si involved, and claims about Si’s net effect on any single pathway should be read with this broader crosstalk, and the still-limited number of studies that measure multiple hormones simultaneously, in mind. By recalibrating rather than simply amplifying hormonal signalling, Si appears to allow treated plants to sustain a more flexible hormonal state that supports both stress tolerance and growth recovery once drought is relieved.

## 8. Cell-Wall Reinforcement and Morpho-Anatomical Adaptation

Si deposits within cell walls, particularly in the epidermis, cuticle, and vascular tissue, reinforcing structural integrity while leaving enough flexibility for turgor-driven cell expansion. These are mechanistically distinct contributions that the literature does not always separate clearly: physical silica polymerisation and deposition is a largely passive, concentration-dependent process, whereas the associated changes in lignin and cellulose biosynthesis are actively regulated at the transcriptional level, and the relative contribution of each to overall wall reinforcement under drought is rarely partitioned experimentally. Supplementation upregulates genes for cellulose, lignin, and xyloglucan biosynthesis, along with genes controlling xylem and phloem histogenesis and patterning, supporting more resilient long-distance water and nutrient transport during drought [12]. The resulting thicker cuticles, more stable stomatal apparatus, and stronger vascular tissue reduce non-stomatal water loss and make water transport more reliable under deficit conditions [42]. In sorghum, enhanced silicification of shoot tissue and root endodermal cells is likewise associated with greater drought resistance; in rice, Si promotes suberin and lignin deposition and casparian band formation in root endodermal and exodermal cells, thought to reduce radial water loss from the root [43]. How Si-reinforced walls retain enough flexibility for turgor-driven expansion while becoming physically stiffer is not mechanistically elaborated in the literature reviewed here; a plausible explanation is that Si deposition is spatially heterogeneous, concentrated in mature epidermal and vascular walls that have largely ceased expanding, while actively growing tissue retains a wall composition, relatively richer in xyloglucan than in crystalline cellulose and silica, that preserves extensibility, but this spatial partitioning has rarely been tested directly. In cereal crops especially, this Si-associated strengthening of vascular and epidermal tissue complements the biochemical mechanisms above rather than substituting for them.

## 9. Transcriptional and Molecular Regulation

The physiological improvements described above are supported by coordinated changes in gene expression. Comparative transcriptomic studies, including RNA-sequencing analyses of drought-tolerant and drought-sensitive lentil genotypes, have shown that Si supplementation alters the expression of genes governing photosynthesis, osmoprotection, antioxidant systems, cell-wall biogenesis, and hormone metabolism, with several hundred to several thousand differentially expressed genes detected depending on genotype and comparison [12]. In rice, Si application has been shown to upregulate transcription factors such as DREB2A and NAC5, which control multiple downstream drought-defence pathways, while also increasing expression of antioxidant-cycle genes including SOD, CAT, APX, and GR homologues [34]. Si nanoparticle formulations have similarly been shown to upregulate stress-related genes such as DREB2, MYB33, and WRKY19 in wheat under drought, indicating convergent transcriptional targets regardless of whether Si is supplied in conventional or nanoparticle form [44,45]. Si also regulates expression of aquaporin genes from both the PIP and TIP families and, in some species, modulates expression of the Si transporter gene Lsi1 itself, indicating feedback regulation within the Si-uptake pathway [46]. Single-nucleus RNA-sequencing in soybean, a Si-excluding legume, has revealed cell-type-specific transcriptional responses to Si treatment, demonstrating that even species with limited bulk Si accumulation possess fine-grained, spatially resolved Si-responsive gene networks [47]. This single-nucleus finding sits within a rapidly maturing methodological landscape: single-cell and spatial transcriptomic approaches are increasingly applied across plant stress biology more broadly, and a recent synthesis of their technical advances and challenges provides a framework for situating cell-type-specific Si responses, such as those reported in soybean, within the wider effort to map spatially resolved stress regulation in plants [48]; integrating this methodological perspective strengthens the case for the temporally and spatially resolved research agenda proposed in Priority 5 below.

## 10. Silicon, Nutrient Homeostasis, and the Rhizosphere

Beyond its direct effects on plant tissue, Si influences the broader root environment in ways that indirectly support drought tolerance. Si application has been shown to alter rhizosphere pH and the availability of phosphorus, potassium, and nitrogen forms, and to modulate the composition of the root-associated microbial community, effects that are increasingly recognised as contributing to overall stress resilience even though their interaction with drought specifically remains comparatively understudied [49]. A recent synthesis situates these effects within an integrated soil–plant–microbiome framework, in which Si-driven changes in rhizosphere chemistry and microbial community composition feed back on shoot-level water and nutrient status rather than acting as a separate, below-ground process [50], and co-inoculation of Si with selected rhizosphere bacteria has itself been shown to improve drought tolerance in a Si-excluding legume, suggesting that plant–microbe and plant–Si effects are not simply additive [51]. Improved nutrient and ion homeostasis under Si supplementation, including favourable potassium-to-sodium ratios and enhanced macro- and micronutrient uptake, supports the maintenance of osmotic and metabolic balance during water deficit, complementing the more direct hydraulic and biochemical mechanisms described above [52]. Precisely how these below-ground changes translate into the shoot-level physiological responses discussed in earlier sections, whether through altered root-to-shoot hormone signalling, changed nutrient delivery to the shoot, or microbially mediated changes in root hydraulic properties, is rarely traced within a single study, leaving this potentially important feedback loop largely inferred rather than demonstrated.

Table 1 summarises representative primary studies illustrating the diversity of crop species, Si delivery methods, and physiological outcomes discussed above.

## 11. Genotype, Dose, and Application-Method Dependency

Si’s benefits are not uniform across genotypes, doses, or delivery methods. A 2025 study in wheat found that the magnitude, and even the direction, of Si’s effect on stomatal conductance and drought resilience depended strongly on cultivar and stress intensity, with some genotypes showing improved water status under Si supplementation while others showed comparatively little benefit under the same drought regime [28]. Field trials in wheat illustrate this dose-dependency directly: pre-plant soil incorporation of diatomaceous-earth-derived Si across a range of 39–117 kg Si ha^−1^ produced a dose-dependent increase in leaf-level water-use efficiency of 32–74% under drought, with the highest rate allowing drought-stressed plants to match the grain yield of well-watered, unsupplemented controls, in a single-season trial [26]. Other field trials illustrate a further, distinct layer of dose- and method-dependency: a two-season, soil-applied field trial using sodium silicate at approximately 20 kg Si ha^−1^, timed to the stem-elongation stage, reported grain-yield increases of 16% and 24% in successive seasons relative to unsupplemented drought controls, illustrating that the magnitude of Si’s effect on yield and water relations depends strongly on application method, soil, foliar, or fertigation, and drought-imposition protocol [2]. These findings indicate that Si should not be regarded as a universally predictable drought remedy, but rather as one whose optimal source, dosage, timing, and expected benefit must be calibrated to genotype, crop stage, and environment [20]. A tomato study illustrates this non-linearity directly: yield, fruit quality, and water-productivity benefits from silicon peaked at an intermediate dose, whether applied as a seed-priming treatment (0.25 mM) or incorporated into soil (300 kg Si ha^−1^), with both lower and substantially higher doses giving smaller gains [53]. Table 2 summarises effective Si doses and application methods reported across a broader set of accumulator and non-accumulator crops, illustrating both the diversity of protocols currently in use and the continued absence of standardised dosing guidance.

## 12. Critical Evaluation of Evidence and Experimental Limitations

The evidence reviewed above is substantial, but several methodological and conceptual limitations constrain how confidently these mechanisms can be generalised. The conflicting reports on Si’s effect on transpiration, for instance, are not resolved by species differences alone; a good part of the discrepancy likely reflects how drought is imposed experimentally. Maintaining pots at a fixed target weight, the most common protocol in controlled studies, requires more frequent irrigation of plants with higher transpiration, which can itself alter the stress severity being compared across treatments and is a poor analogue of progressive field drought [25]. Few of the studies discussed here report soil moisture dynamics in enough detail to rule this out, so some reported Si–drought interactions may be partly an artefact of experimental design rather than a genuine physiological response. A related problem concerns sodium: because Si is most commonly supplied as sodium silicate, the accompanying Na^+^ load is rarely controlled for with an equimolar sodium salt, leaving open the possibility that some reported benefits reflect a Na^+^-mediated osmotic or ionic effect rather than a Si-specific one; only a minority of the primary studies cited here include an appropriate Na^+^ control. Two related questions remain genuinely open. First, whether the divergent transpiration responses documented above reflect a real, species- or genotype-dependent physiological strategy, or are substantially an artefact of target-weight- versus soil-moisture-based drought-imposition protocols, cannot be settled with the current evidence base and is precisely the question Priority 1 below is designed to test. Second, whether the transcriptional and hormonal modules engaged in Si-accumulating grasses are mechanistically identical to those engaged in Si-excluding legumes, or whether they represent convergent but molecularly distinct solutions to the same physiological problem, is likewise unresolved; the single-species designs that dominate this literature cannot distinguish shared mechanism from convergent evolution, which is why Priority 2 below calls for a side-by-side, standardised comparison. The evidence base is also heavily skewed toward a handful of cereal and pseudocereal species, particularly rice, wheat, sorghum, and buckwheat, so how well these mechanisms generalise across the far larger set of Si-excluding dicot crops, including major legumes and horticultural species, remains poorly constrained. Table 2 goes some way toward this: alongside the cereal and legume entries, it now also includes Si–drought studies in several Si-excluding dicot and horticultural crops, including tomato, pepper, potato, faba bean, cowpea, and banana, but this remains a small fraction of the roughly 400 economically important crop species, and coverage of tree and perennial crops in particular stays sparse. Most physiological studies, moreover, span a single drought cycle of days to a few weeks under pot conditions; multi-year, multi-site field validation is essential, given that Si availability and plant Si accumulation both depend strongly on soil pH, texture, and weathering history, yet it remains rare. One notable exception is a recent two-soil, field-scale trial in potato, in which a substantial soil-incorporated Si dose (15 t Si ha^−1^) improved drought tolerance under real field conditions and, as a co-benefit, reduced soil nitrous oxide emissions by roughly 31% [57]; such field-validated, multi-outcome studies remain the exception rather than the norm. A further complication for interpreting soil-applied doses specifically is that drought itself can reduce a plant’s own Si uptake: in barley, water shortage lowered foliar Si content independently of any applied dose [63], meaning the dose that actually reaches a drought-stressed plant may diverge substantially from the nominal amount applied. Transcriptomic evidence is overwhelmingly derived from single- or few-time-point RNA-sequencing snapshots, which can identify differentially expressed genes but cannot on their own establish the temporal order or causal hierarchy of the changes reported; claims that Si acts upstream of a given pathway should therefore be read as hypotheses rather than established sequences. Finally, as with much of the stress-physiology literature, there is a plausible risk of publication bias toward studies reporting a significant Si benefit, which may inflate the apparent consistency of the effects summarised above relative to the true underlying variability. Reporting completeness is also inconsistent across this literature: a subset of the primary studies compiled in Table 2 does not fully specify the Si compound, delivery vehicle, or dissolution mechanism used [15,54], which limits both reproducibility and direct cross-study comparison of dose.

## 13. Mechanistic Overview

**Figure 2:**
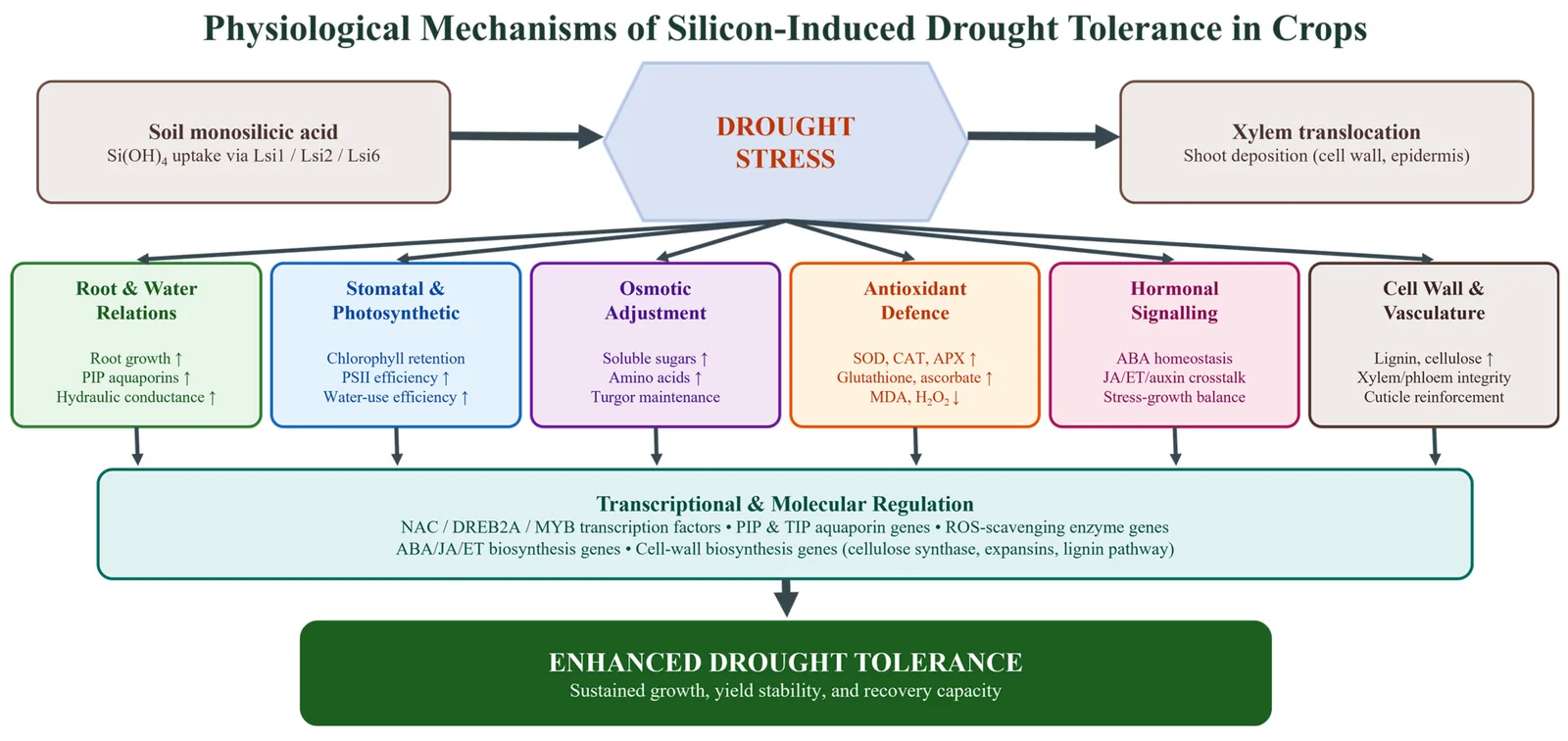
Scheme of the interconnected physiological and molecular mechanisms by which silicon induces drought tolerance in crops. Upward and downward arrows (↑/↓) next to individual components indicate their upregulation/increase or downregulation/decrease, respectively, under silicon-mediated drought tolerance responses.

Si taken up from the soil as monosilicic acid is translocated to the shoot and deposited in cell walls and vascular tissue, while triggering coordinated changes across six physiological domains: root and water relations, stomatal and photosynthetic regulation, osmotic adjustment, antioxidant defence, hormonal signalling, and cell-wall/vasculature reinforcement, all supported by shared transcriptional regulation of aquaporin, antioxidant, hormone-biosynthesis, and cell-wall genes. Confidence in these pathways is not uniform: the aquaporin-mediated hydraulic and enzymatic antioxidant mechanisms rest on relatively direct experimental evidence, including gene-expression silencing, enzyme activity assays, and hydraulic conductance measurements, whereas several of the hormonal and transcriptional linkages described above remain largely correlative, inferred from co-occurring expression changes rather than established through functional validation, and should be weighted accordingly when this scheme is used to guide future work. These pathways converge on an integrated drought-tolerant phenotype marked by sustained growth, more stable yield, and a greater capacity for recovery once water becomes available again, as summarised in Figure 2.

## 14. Conclusions and Future Perspective

Collectively, current evidence indicates that Si alleviates drought stress in crops through a coordinated set of mechanisms rather than any single pathway: enhanced root water acquisition and aquaporin-mediated hydraulic conductance, balanced stomatal and photosynthetic regulation, osmotic adjustment, strengthened antioxidant defence, recalibrated hormonal signalling, structural reinforcement of cell walls and vascular tissue, and coordinated transcriptional regulation across all of these domains. These mechanisms operate to different degrees in Si-accumulating and Si-excluding species, and genotype, dose, application method, and stress intensity all shape the outcome, so turning this mechanistic understanding into practical, genotype-specific Si fertilisation strategies for drought-prone agriculture will need a more targeted research agenda than has typically been pursued.

Building on the conceptual tensions and methodological limitations identified above, this study proposes the following prioritised research agenda for advancing this field over the next five to ten years:

### 14.1. Priority 1: Resolving the Transpiration Paradox

Multi-site field trials that standardise drought-imposition protocols (soil-moisture-based rather than target-weight-based irrigation) and report continuous soil moisture data are needed to determine whether Si’s divergent effects on transpiration reflect genuine physiological plasticity or methodological artefact; parallel measurement of whole-plant hydraulic conductance and leaf-level stomatal kinetics within the same experiment would help separate these possibilities.

### 14.2. Priority 2: Testing Accumulator-Versus-Excluder Equivalence Directly

Side-by-side, standardised comparisons of a Si-accumulating grass and a Si-excluding legume under identical drought, Si-dose, and measurement protocols, ideally within a single multi-omics study, are needed to test whether the same transcriptional and hormonal modules are engaged in both groups or whether distinct, convergent mechanisms are at play.

### 14.3. Priority 3: Establishing Dose-Response and Na^+^-Controlled Trials

Studies that test at least four to five Si concentrations against an equimolar non-silicate sodium control, across both foliar and soil delivery routes, are required to determine true optimal dosing and to separate Si-specific effects from Na^+^-associated effects.

### 14.4. Priority 4: Multi-Year Field Validation

Long-term, multi-site field trials spanning contrasting soil types and rainfall regimes are needed to test whether pot-based mechanistic findings, particularly on root architecture, aquaporin regulation, and yield recovery, translate to realistic agronomic conditions. A specific, testable prediction following from the pot-based evidence reviewed above is that the yield benefit of Si will be significantly larger in coarse-textured, low-Si-availability soils than in fine-textured soils with naturally high plant-available Si, a hypothesis multi-site trials could directly test.

### 14.5. Priority 5: Temporal Resolution of Transcriptional Regulation

Time-course transcriptomic and proteomic sampling across the onset, progression, and recovery phases of drought, combined with functional validation such as CRISPR or RNAi knockouts of candidate transcription factors including DREB2A, NAC5, or MYB33, is needed to move from correlative gene-expression snapshots to causal regulatory models. A specific hypothesis worth testing is whether DREB2A and NAC5 act upstream of the aquaporin and antioxidant gene modules described above, such that their knockout should blunt both the hydraulic and redox components of the Si response simultaneously.

### 14.6. Priority 6: Si–Microbiome–Drought Interactions

Because Si’s effects on the rhizosphere microbiome remain almost entirely uncharacterised under drought specifically, as opposed to salinity or heavy-metal stress, coupled plant–soil–microbiome experiments under controlled drought are needed to determine whether microbiome shifts are a cause or a consequence of improved plant water status. A directly testable version of this question is whether sterilising the rhizosphere microbiome or transplanting a Si-conditioned microbiome into untreated plants reproduces the drought-tolerance benefit normally attributed to direct Si uptake.

Beyond Si alone, a growing body of primary literature points to complementary or additive effects when Si is co-applied with other beneficial or micronutrient elements, spanning selenium, sulphur, zinc, and iron. Under drought specifically, combined Si and Se application alleviated growth and yield loss more effectively than either element alone in rice, by reducing water loss and preserving chlorophyll content [64], and foliar Si–Se nanostructures similarly enhanced antioxidant enzyme activity, relative water content, and membrane stability in drought-stressed strawberry [65]. Combined Si and sulphur foliar fertilisation improved photosynthetic efficiency and water-use productivity in drought-stressed oat [66], and combined Si and zinc application improved chlorophyll retention, osmoprotectant accumulation, and antioxidant defence under drought in Eruca sativa relative to single-element treatments [67]. Under salinity, foliar nano iron-silicon oxide increased triticale grain yield by enhancing chlorophyll content and antioxidant activity while reducing oxidative damage [68], and a recent synthesis situates these element-specific patterns within a broader mechanistic framework spanning heavy-metal, nutrient-deficiency, and osmotic-stress contexts [69]. Whether these co-application benefits extend consistently to the specific transcriptional and hormonal modules discussed above, and whether they hold across the accumulator/excluder distinction central to this review, remains untested and represents a further avenue for the research agenda outlined.

Addressing this agenda would allow the field to move from a descriptive catalogue of Si-associated physiological changes toward a predictive, mechanistically grounded framework for deploying Si as a genotype- and environment-specific drought-management tool.

## Figures and Tables

**Table 1:** Silicon-mediated drought tolerance across different crops, with key findings for each.

Crop	Experiment Type	Si Source/Method	Key Reported Effects/Finding	Reference
Sorghum	Hydroponic (growth chamber)	Hydroponic; aquaporin gene induction	Silicon alleviated salt-induced osmotic stress and upregulated SbPIP1;6, SbPIP2;2, and SbPIP2;6, increasing root hydraulic conductance and water uptake	[22]
Tomato	Hydroponic (growth chamber)	Hydroponic; PEG-induced water stress	Increased root hydraulic conductance; decreased membrane oxidative damage under water stress	[24]
Wheat	Field (rain-manipulation shelters)	Soil-applied (diatomaceous earth) pre-plant; 39–117 kg Si ha^−1^	Field-applied silicon alleviated drought stress and improved water-use efficiency	[26]
Wheat	Field trial	Soil-applied	Improved yield, nutrient uptake, osmotic regulation, and antioxidant responses under field-drought conditions	[2]
Wheat (multi-genotype)	Controlled environment chamber & glasshouse (pots)	Soil-applied, standardised drought trial	Magnitude/direction of stomatal and yield response to silicon strongly genotype- and drought-duration-dependent	[28]
Soybean	Pot (soil-applied, controlled field capacity)	Soil-applied, factorial pot trial	Decreased MDA and H_2_O_2_; increased water-use efficiency under simulated drought	[36]
Rice	Hydroponic (growth chamber)	Hydroponic (2–4 mM Si)	Maintained ROS homeostasis and reduced root cell damage under combined drought + blast-disease stress	[37]
Lentil	Growth chamber (germination assay; PEG-induced)	PEG-simulated osmotic stress	Improved seed germination; regulated osmolytes, hydrolytic enzymes, and antioxidant defence system	[33]
Lentil (Si-excluder)	Pot (growth room); RNA-sequencing	Soil-applied	Feedback-downregulated proline biosynthesis genes; upregulated osmotic-stress and cell-wall genes	[12]
Maize	Soil-column (rain-shelter, semi-controlled outdoor)	Foliar application	Improved shoot/root growth and photosynthesis; JA-signalling shifts linked to photosynthetic preservation	[30]
Peach (seedlings)	Pot (greenhouse; PEG-simulated drought)	Root-applied (Na_2_SiO_3_ solution)	Coordinated regulation of hormone, amino-acid, and sugar metabolism; improved WUE and root development	[41]
Common buckwheat	Pot (Green house)	Foliar application	Improved gas exchange and PSII function; accelerated recovery from combined drought + waterlogging	[31]
Common buckwheat	Pot (Green house)	Foliar application	Stabilised photosynthetic performance and enhanced productivity under drought	[32]
Soybean (Si-excluder)	Hydroponic (growth chamber); not drought-specific	Hydroponic; single-nucleus RNA-sequencing	Cell-type-specific transcriptional responses to silicon despite low bulk Si accumulation	[47]

**Table 2:** Effective silicon doses and application methods reported in drought-tolerance studies across accumulator and non-accumulator crops.

Crop	Effective Si Dose/Application Method	Reference
Wheat	Soil-applied (diatomaceous earth, pre-plant); 39, 78, and 117 kg Si ha^−1^ (low/med/high); achieved by adding 2.81, 5.62, and 8.43 g of Agrisilica, respectively	[26]
Wheat (multi-genotype)	1.8 mM sodium metasilicate (Na_2_SiO_3_·H_2_O) applied through compost, 3.6 mM sodium chloride (NaCl) was used for the 0 mM Si treatment (to balance the Na+ ions)	[28]
Wheat	Soil-applied sodium silicate, ~20 kg Si ha^−1^, applied at stem elongation; two-season field trial, grain yield +16% (yr 1) and +24% (yr 2) vs. unsupplemented drought controls	[2]
Rice	Na_2_SiO_3_·9H_2_O was applied hydroponically at 0, 1, 2, 4, and 6 mM concentration	[37]
Maize	Soil-applied, 50 mg Si kg^−1^ soil	[54]
Sorghum	1.67 mM Na_2_SiO_3_ through hydroponic system	[22]
Chickpea	Foliar, 50 mg Si L^−1^, applied as silica gel	[15]
Chickpea	Exogenous application of 1.5 mM Si as CaSiO_3_ through irrigation system	[14]
Common bean	0.1–2.0 mmol of Na_2_SiO_3_ in hydroponic nutrient solution	[16]
Mung bean	30–90 mg Na_2_SiO_3_ L^−1^ applied through foliar spray	[17]
Lentil	1 and 4 mM K_2_SiO_3_ applied through soil drench	[51]
Soybean	100, 200, and 300 kg of Na_2_SiO_3_ ha^−1^ applied through soil (at sowing)	[36]
Cotton	Monosilicic acid (20% Si) applied through soil drench (60 kg ha^−1^) and 1 mM through seed priming	[55]
Peach (seedlings)	Na_2_SiO_3_ applied through hydroponic culture (0.06 mM/L) whereas 0.6 mM was applied through root drench (in quartz sand system)	[41]
Common buckwheat	Foliar spray, 1 mM sodium metasilicate nonahydrate (Na_2_SiO_3_·9H_2_O) sprayed twice—once at the start of the 15-day stress period and once at the start of the 15-day recovery phase	[31]
Tomato	Seed priming with monosilicic acid (MSA), 0.25 mM; or soil-incorporated MSA, 300 kg Si ha^−1^ (screened across 0–0.5 mM priming and 0–600 kg ha^−1^ soil-dose ranges)	[53]
Pepper	Exogenous Na_2_SiO_3_ at 1 mM concentration, method not clear (likely soil applied)	[56]
Potato	Soil-incorporated amorphous silica or diatomaceous earth, 15 t Si ha^−1^, field-scale trial (also reduced soil N_2_O emissions by ~31%)	[57]
Sugarcane	2.5 mM sodium and potassium silicate, stabilized with sorbitol (pH-adjusted to 5.5 ± 0.2), two combined routes applied together: fertigation and foliar spray	[58]
Rapeseed (Brassica napus)	1 mM SiO_2_, semi-hydroponic uptake. Seedlings were grown in Petri dishes lined with six layers of filter paper (moistened with diluted Hyponex nutrient solution), and Si was directly added to it.	[59]
Faba bean	Foliar spray, potassium silicate at 100 ppm, applied three times (30, 45, and 60 days after sowing); tested alongside chitosan and tryptophan anti-transpirants	[60]
Cowpea	Foliar spray, 300 mg L^−1^ of Sifol^®^ powder (Diatom Mineração Ltda., Mogi das Cruzes, São Paulo, Brazil), a commercial silicon source containing ~42% silicon in the form of silicic acid (H_4_SiO_4_) on the 22nd and 43rd days after sowing (the same factorial trial also tested 890 mg L^−1^ L-methionine alone and combined with the Si treatment, on the same schedule)	[61]
Banana	SiO_2_ nanoparticles: 150 mg L^−1^ in vitro (culture medium) or 600 mg L^−1^ foliar spray (greenhouse, 250 mL/plant, twice monthly)	[62]

## Data Availability

No new data were created or analyzed in this study. Data sharing is not applicable to this article.
